# Incidental Removal of an Aspirated Tooth During Routine Endotracheal Suctioning: A Case Report

**DOI:** 10.3390/healthcare14162462

**Published:** 2026-08-10

**Authors:** Se Kwang Oh

**Affiliations:** 1 Department of Emergency Medicine, Chungnam National University Sejong Hospital, Sejong 30099, Republic of Korea; skoho@cnuh.co.kr; Tel.: +82-44-995-3021; 2 Department of Emergency Medicine, College of Medicine, Chungnam National University, Daejeon 34134, Republic of Korea

**Keywords:** foreign bodies, tooth, bronchoscopy, airway management, aspiration, intubation, intratracheal

## Abstract

Background/Objectives: Foreign body aspiration is a potentially life-threatening emergency that commonly requires bronchoscopic removal. Dental aspiration in elderly patients may occur following trauma, seizures, or altered mental status and can lead to airway obstruction and respiratory complications. We report a rare case of simultaneous airway and esophageal foreign bodies caused by tooth dislodgement after a generalized seizure in an elderly patient. Methods: An 85-year-old man presented to the emergency department with decreased consciousness and recurrent generalized seizures. Chest computed tomography revealed two tooth-like foreign bodies located in the right main bronchus and the mid-esophagus. Endotracheal intubation was performed because of persistent altered mental status, oral bleeding, and retained airway secretions. Although emergency bronchoscopy was considered necessary, it was not immediately available overnight. Repeated endotracheal suctioning was performed while maintaining airway protection and oxygenation. Results: Approximately 12 h later, a tooth-like foreign body was recovered attached to the tip of the suction catheter immediately after withdrawal during routine endotracheal suctioning, suggesting spontaneous proximal migration before its removal. Follow-up imaging confirmed disappearance of the bronchial foreign body, whereas the esophageal foreign body was subsequently removed endoscopically. The patient remained clinically stable without procedure-related complications. Conclusions: This case demonstrates that routine endotracheal suctioning may occasionally facilitate the incidental removal of a small, mobile tracheobronchial foreign body under exceptional circumstances. To our knowledge, reports of aspirated teeth being removed solely by routine endotracheal suctioning without bronchoscopic intervention are extremely rare. However, bronchoscopy remains the standard treatment for tracheobronchial foreign body removal, and suction-assisted removal should be regarded only as an incidental event rather than an alternative therapeutic strategy.

## 1. Introduction

Foreign body aspiration within the airway is a potentially life-threatening emergency that can occur not only in children but also in elderly patients. Rigid objects such as teeth are of particular concern, as they may lead to airway obstruction, hypoxemia, and secondary complications including pneumonia. Prompt recognition and timely management are therefore essential [1,2].

Dental foreign bodies may arise in a variety of clinical settings, including trauma, seizures, altered mental status, or during airway manipulation. [3,4]. Although dental aspiration in adults is relatively uncommon, published studies have demonstrated that bronchoscopic retrieval is successful in the vast majority of cases [5,6]. In most cases, removal via bronchoscopy is considered the standard and definitive approach [7]. However, in situations where the patient is hemodynamically unstable or when immediate access to bronchoscopic intervention is limited, alternative management options may be constrained.

Although there have been occasional reports of spontaneous migration or expulsion of airway foreign bodies induced by coughing or positive pressure ventilation, successful removal by suction alone following endotracheal intubation has rarely been described [8,9].

Here, we report a rare case in which an aspirated tooth was unexpectedly removed during repeated endotracheal suctioning following intubation without bronchoscopic intervention in an elderly patient presenting with decreased consciousness and seizures.

## 2. Case Presentation

An 85-year-old man was brought to the emergency department after a sudden loss of consciousness while in a sauna. On arrival at the emergency department, the patient’s vital signs were as follows: blood pressure 101/59 mmHg, heart rate 175 beats/min, respiratory rate 45 breaths/min, body temperature 39.2 °C, and oxygen saturation 100% while receiving oxygen at 15 L/min via a reservoir mask administered by emergency medical services. His mental status was depressed, and he was responsive only to painful stimuli. He subsequently developed a generalized tonic–clonic seizure, which was treated with intravenous midazolam (3 mg). Mild-to-moderate oral bleeding and retained oral secretions were noted following the seizure. Physical examination revealed the loss of two anterior teeth; however, neither the missing teeth nor any foreign body could be identified within the oral cavity.

**Figure 1:**
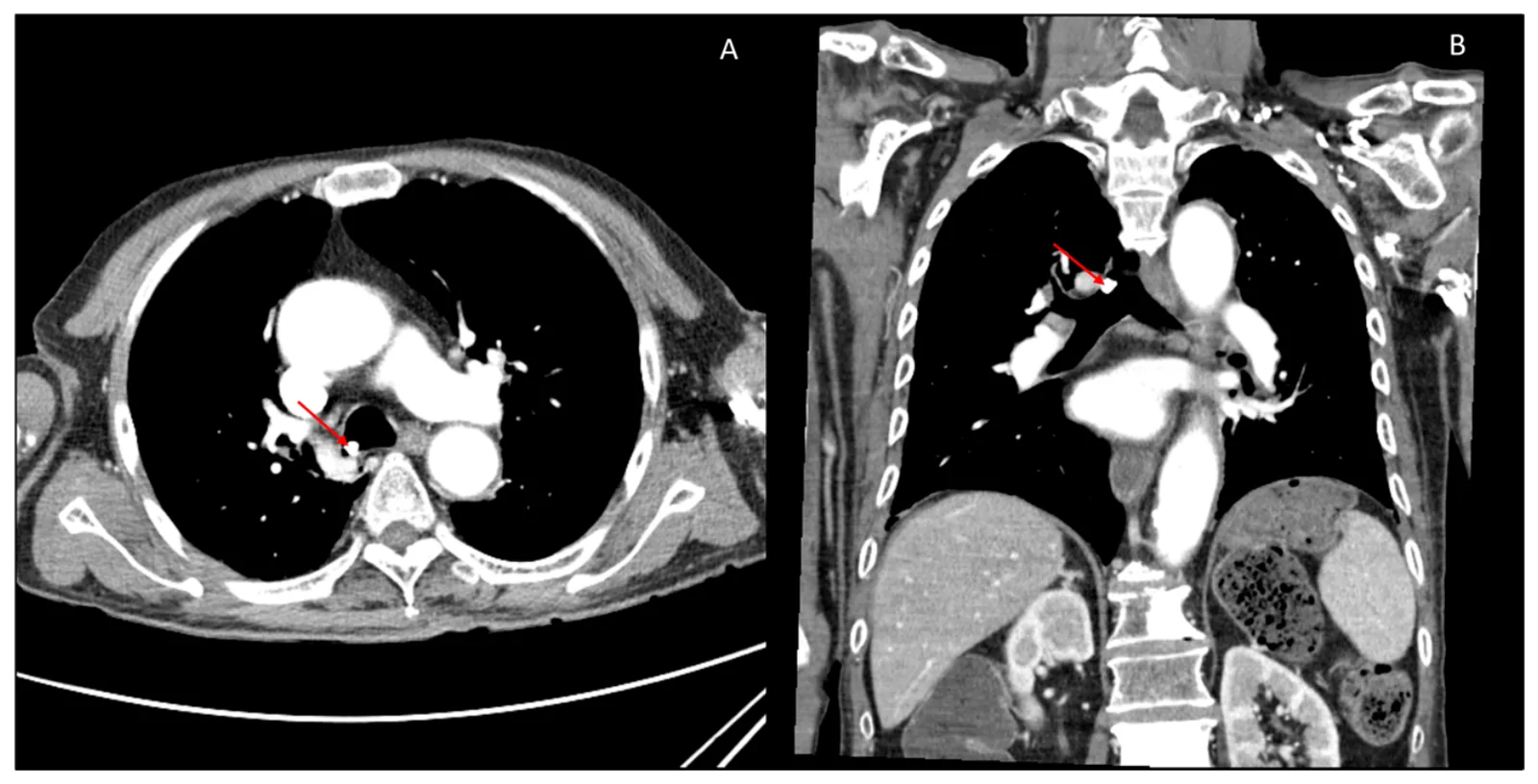
Initial contrast-enhanced chest computed tomography. (**A**) Axial CT image demonstrating a tooth-like radiopaque foreign body in the right main bronchus (red arrow). (**B**) Coronal reconstructed CT image demonstrating the same foreign body in the right main bronchus (red arrow).

**Figure 2:**
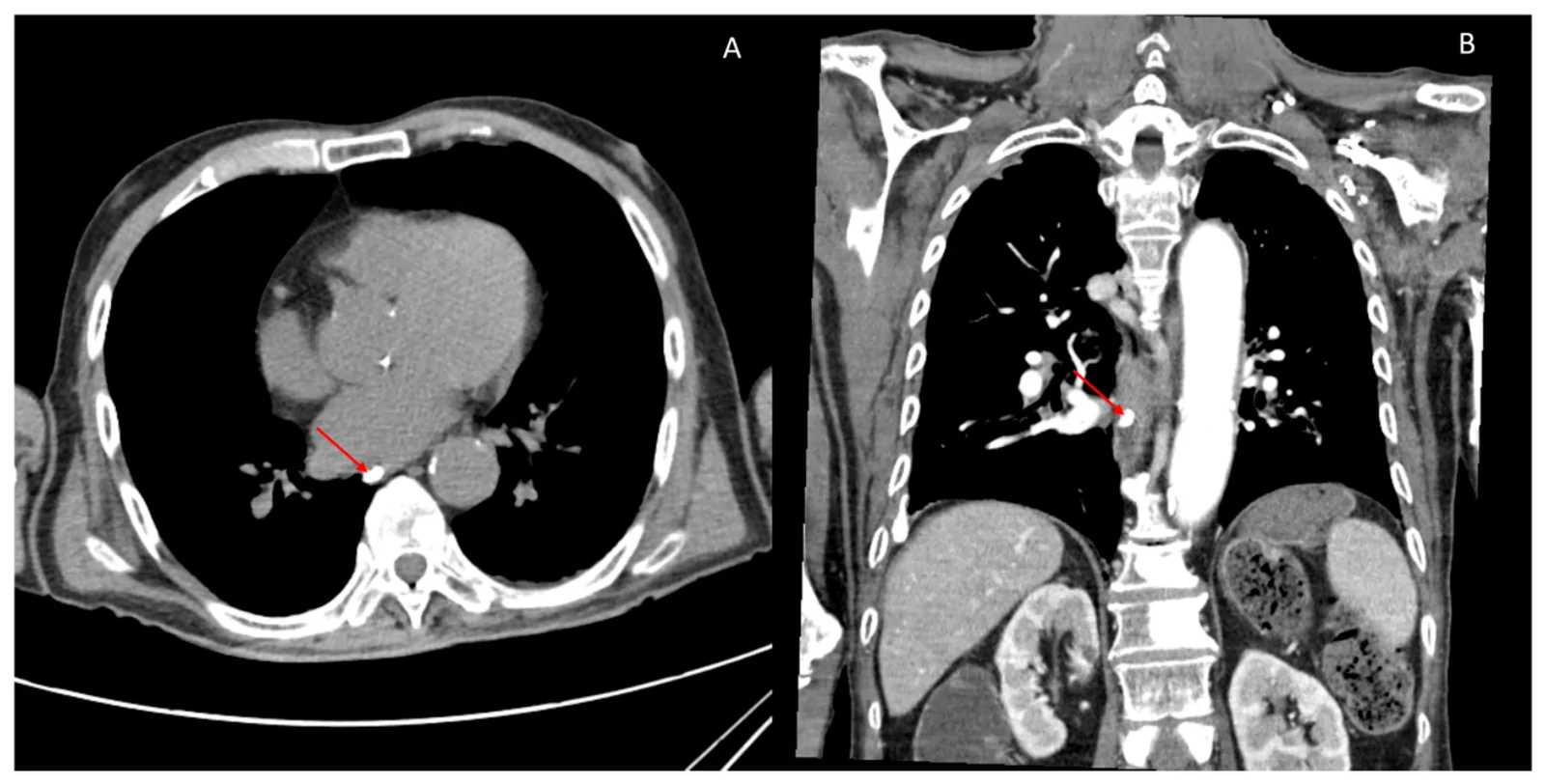
Chest computed tomography demonstrating a tooth-like radiopaque foreign body in the lower esophagus. (**A**) Axial CT image. (**B**) Coronal reconstructed CT image. The red arrows indicate the suspected tooth-like foreign body.

Given the presence of seizure activity accompanied by fever and suspected pneumonia, a series of diagnostic evaluations, including chest computed tomography (CT), were performed. Although no significant radiologic evidence of pneumonia was observed, CT imaging revealed two radiopaque foreign bodies consistent with teeth, located in the right main bronchus and the lower esophagus, respectively (Figure 1 and Figure 2).

During observation in the emergency department, the patient experienced an additional seizure and remained in a stuporous state without improvement in consciousness. Because of the persistent depressed mental status, mild-to-moderate oral bleeding, and retained oral secretions, endotracheal intubation was performed using a 7.5 mm internal diameter endotracheal tube to secure the airway and prevent aspiration. Mechanical ventilation was not initiated because spontaneous respiration and oxygenation remained adequate after intubation.

Emergency bronchoscopic removal of the airway foreign body was considered necessary. However, because the event occurred during overnight hours, no bronchoscopist was available to perform emergency bronchoscopy at our institution. Transfer to another hospital was also considered; however, no nearby referral center was able to provide emergency bronchoscopic intervention during the night. Following endotracheal intubation, the patient remained hemodynamically stable and required only minimal supplemental oxygen. Therefore, conservative airway management with close observation was continued in the emergency department while bronchoscopic availability was reassessed the following morning. To manage ongoing airway secretions, routine endotracheal suctioning was performed using a standard open suction system with a 14-Fr suction catheter advanced to the distal end of the endotracheal tube. Suctioning was performed as clinically indicated, approximately every 2–3 h. The suction pressure was not documented in the medical record.

**Figure 3:**
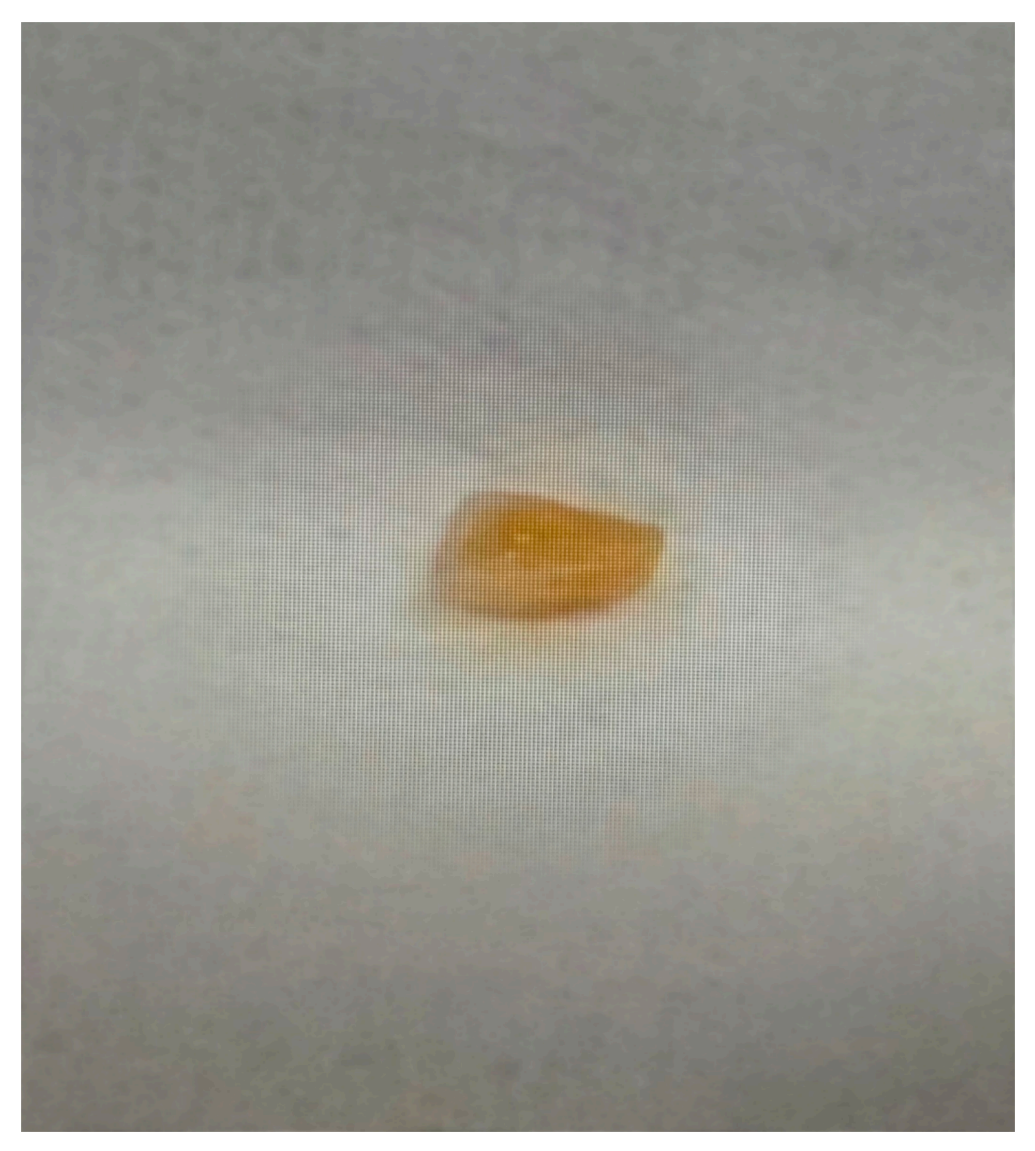
Photograph of the removed tooth recovered attached to the tip of the suction catheter during routine endotracheal suctioning (approximately 7 mm × 5 mm).

**Figure 4:**
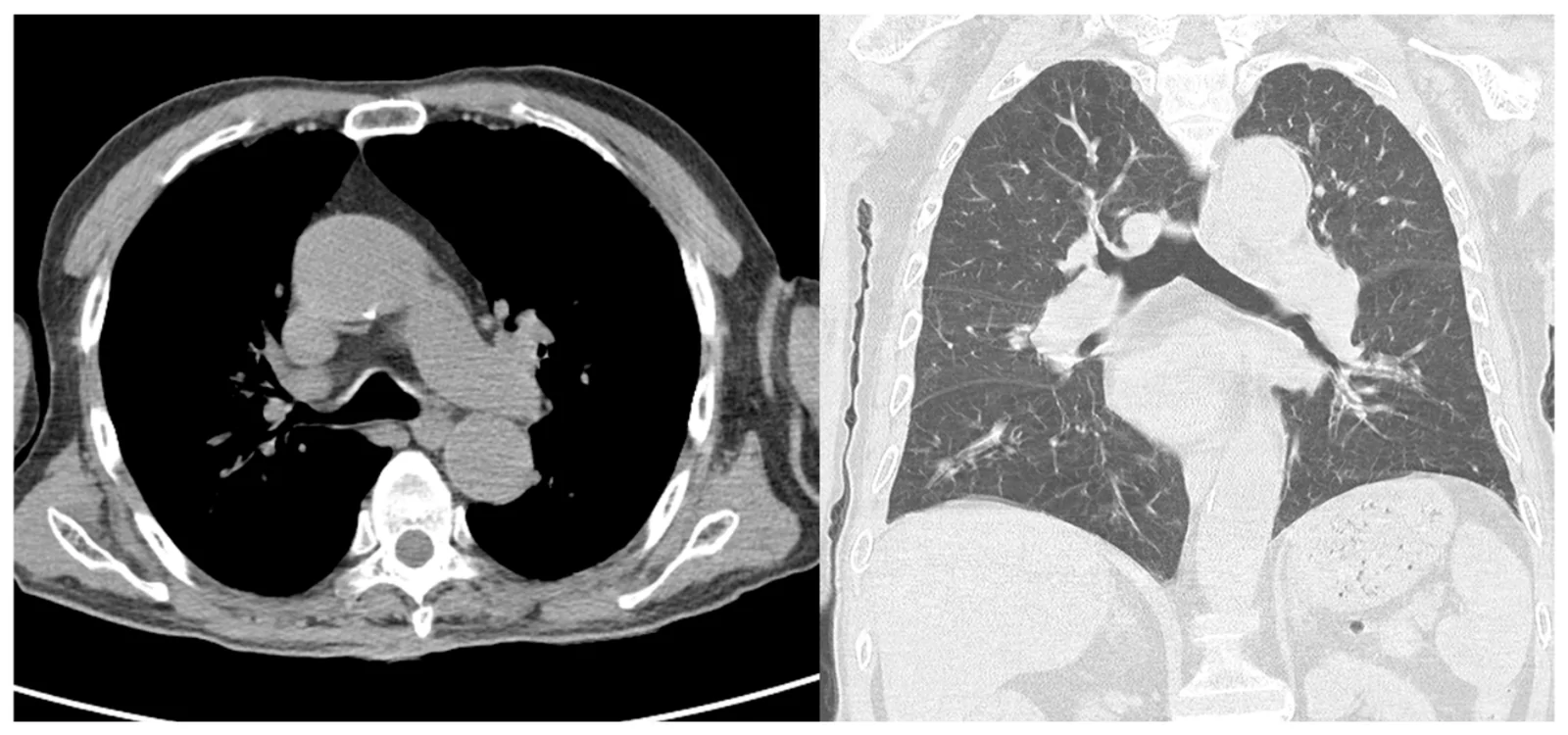
Follow-up chest computed tomography demonstrating disappearance of the previously identified foreign body in the right main bronchus after routine endotracheal suctioning on axial and coronal CT images. No obvious associated pulmonary abnormalities, including pneumothorax or atelectasis, were identified.

Approximately 12 h after presentation, during routine endotracheal suctioning, the aspirated tooth (approximately 7 mm × 5 mm) was recovered attached to the tip of the suction catheter immediately after the catheter was withdrawn from the endotracheal tube (Figure 3). It was presumed that the bronchial foreign body had been dislodged and removed during suctioning. A follow-up chest CT was performed to confirm this finding, which demonstrated that the previously observed foreign body in the right main bronchus was no longer present (Figure 4), with no evidence of pneumonia, atelectasis, or other respiratory complications. Throughout and after the suctioning event, the patient remained hemodynamically stable without respiratory deterioration, including no worsening hypoxemia or increased oxygen requirement.

The esophageal foreign body remained unchanged and was subsequently managed by esophagogastroduodenoscopy. Because of its small size, it was gently advanced into the stomach using the endoscope without evidence of esophageal mucosal injury. No respiratory complications, including respiratory distress, hemoptysis, pneumonia, or pneumothorax, developed during hospitalization. Following these interventions, the patient’s mental status gradually improved, and the endotracheal tube was successfully removed. After stabilization and recovery of consciousness, the patient was transferred to another hospital where he had been receiving treatment for prostate cancer.

## 3. Discussion

This case describes a rare situation in which tooth loss following a generalized seizure resulted in simultaneous foreign bodies within both the airway and the esophagus. During generalized tonic–clonic seizures, strong contraction of the masticatory muscles and traumatic contact between the upper and lower teeth may occur. In elderly patients, particularly those with poor dentition or underlying periodontal disease, these forces may increase the likelihood of tooth dislodgement. Once detached, teeth may either be swallowed or aspirated, leading to esophageal or tracheobronchial foreign bodies. Although dental aspiration has been reported in adults, simultaneous involvement of both the airway and esophagus appears to be relatively uncommon [1,2,3].

Removal of airway foreign bodies is generally achieved through bronchoscopic intervention, which remains the standard and definitive management strategy [4,7,10]. Bronchoscopy is especially important when the foreign body is large, rigid, sharp, or located in the distal bronchial tree. In the present case, bronchoscopic removal was initially considered necessary after identification of the foreign body within the right main bronchus. However, immediate bronchoscopic intervention was not available because the event occurred outside routine bronchoscopy service hours. Under these circumstances, the patient underwent endotracheal intubation for airway protection and oxygenation, followed by close observation and repeated suctioning through the endotracheal tube. Unexpectedly, the bronchial foreign body was recovered following repeated endotracheal suctioning, although the exact mechanism of removal remains uncertain.

Endotracheal suctioning is primarily intended for the removal of airway secretions and is not recommended as a standard method for foreign body extraction. Nevertheless, under selected conditions, suctioning may contribute to removal of small and mobile airway foreign bodies. If the object is located near the proximal airway or close to the tip of the endotracheal tube, it may become dislodged during coughing, airway pressure changes, or catheter suctioning [10,11]. In the present case, the foreign body was located within the right main bronchus and may have migrated proximally as a result of coughing, airway pressure changes, airway manipulation, repeated suctioning, or a combination of these factors, eventually allowing its recovery through the endotracheal tube.

Despite this outcome, such an approach should be interpreted with caution. Repeated blind suctioning attempts may push the foreign body further into the distal airway, cause mucosal injury, or even precipitate acute airway obstruction [4,7,10]. In particular, suction-based removal is unlikely to be effective for large, sharp, or distally impacted foreign bodies and may instead increase procedural risk. Therefore, indiscriminate or excessively deep suctioning maneuvers should be avoided.

This case may provide practical clinical insight into situations in which immediate bronchoscopy is not readily available. In such settings, securing the airway and maintaining adequate oxygenation should remain the primary priorities. Careful assessment of foreign body location through imaging studies is also essential. Routine endotracheal suctioning should be performed only when clinically indicated for airway secretion management. In rare circumstances, incidental recovery of a small airway foreign body may occur during this routine procedure. However, blind endotracheal suctioning should not be regarded as a therapeutic alternative to bronchoscopic foreign body removal and must never delay definitive bronchoscopic management whenever available. Another limitation of this case is that follow-up bronchoscopy was not performed after recovery of the foreign body. Although follow-up chest CT confirmed disappearance of the bronchial foreign body, bronchoscopy would have allowed direct assessment of mucosal injury, bleeding, edema, residual fragments, or airway inflammation. Therefore, minor residual airway injury could not be completely excluded.

The simultaneous presence of airway and esophageal foreign bodies in the present case also highlights an important diagnostic consideration. In patients with seizures, facial trauma, or altered mental status, unexplained missing teeth should prompt careful evaluation of both the airway and the gastrointestinal tract. Although airway foreign bodies require immediate attention because of the risk of respiratory compromise, swallowed teeth may also cause clinically significant complications, including esophageal obstruction or gastrointestinal injury, and therefore should not be overlooked. Early imaging evaluation of both systems may facilitate timely diagnosis and appropriate management when simultaneous foreign bodies are present.

## 4. Conclusions

In conclusion, suctioning through an endotracheal tube may occasionally contribute to removal of small airway foreign bodies under specific conditions, but it should not be regarded as a substitute for bronchoscopic intervention. Bronchoscopy remains the standard treatment for tracheobronchial foreign body removal, and the present case should be interpreted as an unusual and limited clinical experience rather than an alternative therapeutic strategy.

## Data Availability

The original contributions presented in this study are included in the article. Further inquiries can be directed to the corresponding author.
